# From Harm to Hope: Tackling Microplastics’ Perils with Recycling Innovation

**DOI:** 10.3390/molecules30122535

**Published:** 2025-06-10

**Authors:** Irene Dini, Andrea Mancusi, Serenella Seccia

**Affiliations:** 1 Department of Pharmacy, University of Naples Federico II, Via Domenico Montesano 49, 80131 Napoli, Italy; seccia@unina.it; 2 Department of Food Microbiology, Istituto Zooprofilattico Sperimentale del Mezzogiorno, Via Salute 2, 80055 Portici, Italy; andrea.mancusi@izsmportici.it

**Keywords:** food pollutants, microplastics, nanoplastics, POP, analytical test, circular economy, plastic waste valorization

## Abstract

This review examines how plastics break down into dangerous pollutants like microplastics, nanoplastics, and persistent organic pollutants (POPs) that can contaminate the environment, make their way into the human food chain, and provoke toxicological effects in humans. According to the reviewed literature, new biomarkers associated with their exposure should be identified, and new methods for detecting them in the environment and in food should be developed and validated. It would also be interesting to improve research on the interaction between micro- and nanoplastics and human cells, their impact on DNA, and their long-term health effects. Promoting sustainable practices and adherence to the 3R strategies (reduce, reuse, and recycle) to transform hazardous waste into valuable resources is crucial to protecting public health from dangerous contaminants as we wait on the development of new diagnostic methods and more stringent legislation.

## 1. Introduction

Some plastic materials can contaminate the environment with POPs (such as dioxins and furans) [1] and impact carbon emissions [2]. Improper plastic disposal can damage ecosystems and human health, not only because of the adverse effects linked to their existence [3] but also because plastic waste can accumulate and transport heavy metals, pesticides, and other persistent organic pollutants [4]. In the ocean, plastic debris is fragmented into microplastics (MPs) and nanoplastics (NPs) [5], which can adhere to marine salt or be ingested by marine organisms, subsequently entering the food chain and building up in humans, leading to uncertain consequences [6]. Marine plastic pollution increases atmospheric CO_2_ levels, diminishing the ocean’s ability to sequester carbon [7]. The significant global crisis due to plastic waste is exacerbated by gas emissions from plastic incineration [8,9]. This paper reviewed papers discussing what happens to plastics when they are disposed of in different ecosystems (terrestrial, marine, air), how their derivatives can affect human health, the methods by which it is possible to determine their derivatives in traces, and sustainable approaches designed to reduce plastic waste.

## 2. Plastics and Micro- and Nanoplastics

### 2.1. Plastics Classification

**Figure 1:**
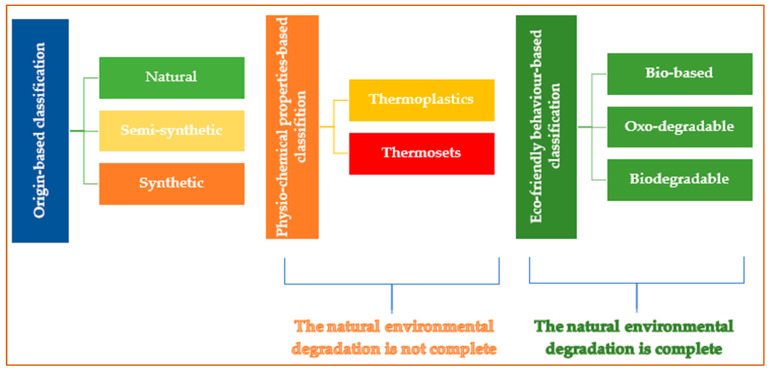
Plastics classification.

Plastics are polymeric molecules with high molecular weight [10] and with additives such as plasticizers, flame retardants, stabilizers, pigments, and lubricants. The additives do not chemically bond with plastics except for some reactive organic additives that integrate into the polymer chain [11]. “Plastics” refers to substances readily molded into diverse shapes and sizes. It originates from the Greek word “plasticos” [12]. Plastics can be classified based on origin, temperature-dependent behavior, and preservative techniques (Figure 1).

Origin-based classification divides plastics into natural, semi-synthetic, and synthetic subdomains. Natural polymers such as chitin, lignin, and starch are derived from substances like horn, amber, and tortoiseshell, while synthetic polymers, including silicone, polyethylene, polystyrene, and nylon, are produced from petrochemicals [13] (Table 1).

Semi-synthetic plastics are created by altering natural polymer materials like cellulose [28].

Plastics can be classified as thermoplastics based on their physicochemical properties and thermosets. The thermoplastics are suitable for recycling. They can soften upon heating, can melt, and can be reshaped multiple times [29].

Thermosets are materials that cannot be remelted after being formed. They retain their structural integrity when subjected to heat [30]. It is essential to understand that neither thermoplastics nor thermosets undergo complete natural environmental degradation.

On the contrary, bio-based, oxo-degradable, and biodegradable plastics are expected to degrade in the environment, yet their ability to biodegrade is a subject of debate. Biodegradable plastics can produce MPs since their degradation is designed for specific conditions, like composting, and they face challenges in breaking down in natural environments like soil or ocean ecosystems. Various factors, such as temperature, microbial presence, oxygen levels, and environmental conditions, can affect their degradation [31]. Numerous bioplastics produce residues (including POPs), undermining their assertions of being sustainable [32]. Additionally, the biodegradation of bioplastics may present certain disadvantages. As they decompose, they can release nutrients that can lead to eutrophication, and the availability of microorganisms responsible for biodegradation to higher-trophic-level organisms might decrease, potentially disrupting food webs and resulting in the accumulation of toxins [33]. Thus, properly managing biodegradation in complex ecosystems is vital to weigh the benefits against potential risks [34].

Bio-based plastics like bio-polyethylene (bio-PE), polyethylene terephthalate (bio-PET), and bio-polyamides (bio-PA) are derived from renewable sources such as plants, corn cellulose, and starch [31]. Because they come from sustainable materials, they have a reduced carbon footprint and improved circularity. However, scientific evidence concerning the biodegradability and sustainability of these new materials is still lacking [35].

Oxo-degradable plastics comprise polyethylene mixed with TDPA™ (London, UK) to improve polymer chains. They allow much fragmentation but do not bring the compound down to its elementary base [36]. Oxo-degradable plastics are classified into hydro-degradable (which break down from hydrolysis) and photodegradable plastics (which break down from UV radiation) [37].

Bioplastics (e.g., PLA, PHA, polylactide, and polycaprolactone) are polymers that form biogases and biomass when they are subjected to microbial degradation [38].

### 2.2. Micro- and Nanoplastics

Under ecological stress (ultraviolet rays and digestion) and biotic processes, synthetic plastics can decompose into microplastics (0.1 µm≤diameter≤ 5000 µm) and nanoplastics (diameter ≤ 1000 nm) [10]. The fibers and fragments are the most frequently encountered forms in seawater [39]. Elements leading to the presence of MPs and NPs in the environment include urban dust, markings on road surfaces, and beauty products [40].

Microplastics can be divided into primary microplastics (i.e., microbeads in personal care items, plastic pellets used in industry, and synthetic fibers from textiles released during washing), which are released directly into the environment, and secondary microplastics (i.e., plastic bottle fragments, fishing net fibers, and vehicle tire abrasion), which are produced from the degradation of larger plastic items [41]. The primary sources of secondary plastics are laundering textiles, made from mixed synthetic fibers [42], and the wear of car tires [43].

### 2.3. Degradation of Plastics

Six processes can break down plastic materials: photodegradation, thermal (or oxidative) degradation, ozone degradation, mechanochemical degradation, catalytic degradation, and biodegradation.

Photochemical degradation reactions occur on the polymer surface. Ultraviolet radiation breaks C–C bonds by forming esters, aldehydes, and propyls [44].

Thermal degradation reactions affect the entire polymer, initiating from the weakest bonds. They necessitate the presence of ultraviolet light and elevated temperatures [45]. Ozone degrades polymeric materials by generating reactive oxygen species [46].

The mechanochemical process can reduce the weight of the molecules by ultrasonic irradiation (which generates radical reactions on the chain side) or mechanical stress.

Catalytic degradation process reduces polyolefins into oils and gas [12].

Biodegradation consists of natural and synthetic plastic degradation by bacteria, fungi, and *Actinomycetes*. The process results in low-molar-mass molecules such as acids, terpenes, aldehydes, water, and gases such as methane, carbon dioxide, and nitrogen [47]. It can happen in aerobic and anaerobic modes. Aerobic biodegradation produces CO_2_ and H_2_O. Anaerobic processes generate CO_2_ and CH_4_ [48].

**Figure 2:**
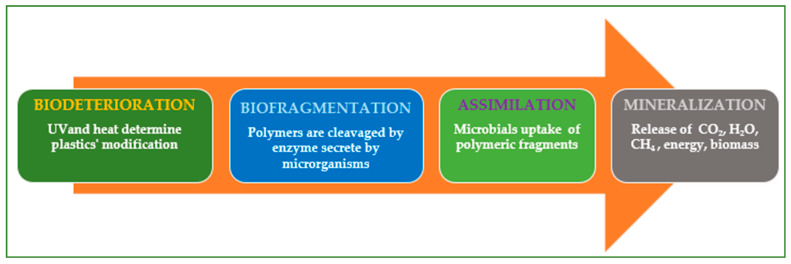
Polymeric materials’ biodegradation.

Polymeric materials’ biodegradation comprises biodeterioration, biofragmentation, assimilation, and mineralization (Figure 2).

Biodeterioration refers to the process in which mechanical, physical, and chemical stresses alter the plastic waste (the decline of the plastic materials can be observed without the aid of instruments). Biofragmentation refers to how microbial enzymes (e.g., lipase, manganese peroxidase, esterase, amidase, and laccase) decrease the polymers’ molecular weight [49]. Assimilation refers to how microorganisms employ polymers as carbon and nitrogen sources during aerobic respiration, anaerobic respiration, and fermentation [47].

Mineralization refers to the process in which organic material transforms into minerals, water, and gases such as methane (CH_4_), carbon dioxide (CO_2_), and nitrogen compounds. Mineralization concludes when microorganisms transform all carbon atoms into carbon dioxide [50]. Table 2 presents a side-by-side analysis of various techniques employed in plastic degradation, offering valuable insights into their effectiveness and applications.

Microplastic type, morphology, dimensions, and coloration provide significant insights to identifying the microplastics’ origin [51]. Fragments with sharp edges are recent entries into marine environments or are derived from the recent disintegration of more extensive plastic materials. Conversely, fragments with smooth edges originate from older materials that have undergone continuous abrasion by other particles or sediments [52].

The standard approach for evaluating the biodegradability of synthetic polymers involves measuring CO_2_ emissions from soils that contain biodegradable plastics compared to those that do not. Because the breakdown of soil organic matter might lead to overestimating their levels, stable carbon isotopes can be measured to distinguish between CO_2_ resulting from plastic degradation and soil organic matter’s mineralization [53].

## 3. Plastics’ Impact on the Ecosystem

Nearly 80% of plastic waste in the past 75 years has ended up in landfills or natural environments, where it can remain for decades or centuries, slowly breaking into smaller pieces. The kind of plastic, how much there is, its additives, and its shape can harm ecosystems differently [54].

### 3.1. Microplastics’ Impact on the Terrestrial Ecosystem

**Figure 3:**
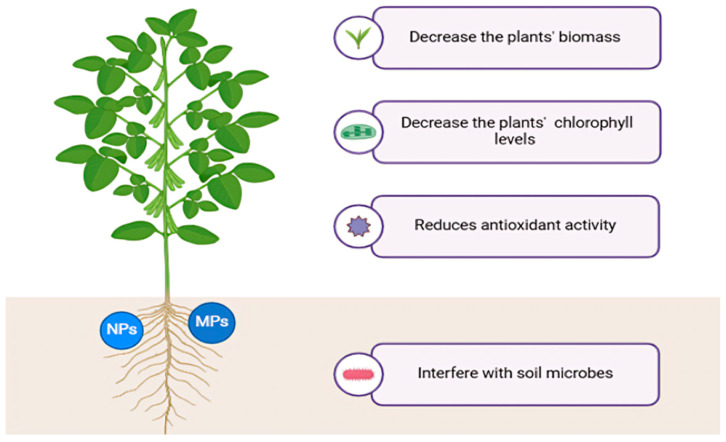
Impact of plastic derivatives on plants.

MPs can influence soil pH, water retention capacity, and conductivity [55,56,57]. They affect plant tissues’ elemental composition and oxidative stress response and decrease plants’ biomass and chlorophyll levels [58,59]. MPs can interfere with soil microbes’ metabolic processes [60] and improve the growth of ones able to metabolize the microplastic, such as *Aeromicrobium*, *Mycobacterium*, and *Amycolatopsis* [61] (Figure 3).

Microplastics can carry other pollutants that can adhere to their surfaces using hydrophobic interactions, non-covalent bonding, electrostatic attraction (e.g., van der Waals forces), and microporous mechanisms [62].

### 3.2. Microplastics’ Impact on the Aquatic Ecosystem

Aquatic ecosystems collect microplastics from wastewater treatment facilities, precipitation, soil erosion, sewer overflow events, and agricultural plastic materials. The plastic collection can be influenced by flow rate, hydraulic gradient, retention time, water volume, and seasonal changes [63]. Freshwater ecosystems are responsible for 80% of the MPs in marine environments. MPs can intensify the melting of Antarctic glaciers by affecting their ability to absorb light [64]. MPs in aquatic ecosystems can end up in the sediment from minor water bodies to larger aquatic ecosystems [65]. MPs can move human-made pollutants, including organic polycyclic aromatic hydrocarbons (PAHs), organochlorine compounds (OCs), and polychlorinated biphenyls (PCBs), and inorganic pollutants like cadmium (Cd), nickel (Ni), chromium (Cr), and lead (Pb) between various environments. Human-made pollutants can be categorized into regulated priority contaminants and Contaminants of Emerging Concern (CECs). CECs are unregulated due to inadequate data demonstrating their environmental hazards. This second category includes chemicals in personal care items, pharmaceuticals, organophosphorus flame retardants, and endocrine-disrupting substances [66].

High temperatures, acidic pH, and salinity can increase pollutants’ adsorption on plastic surfaces [67].

Elevated temperatures amplify microplastics’ negative effect on digestive functions and increase the metabolic demand for aerobic respiration [68].

The pH level can influence electrostatic interactions [69]. When the pH exceeds the MPs’ zero charge point, the MPs’ surfaces gain a negative charge. Conversely, if the pH surpasses the organic pollutants’ acid dissociation constant, the pollutants become deprotonated and anionic, resulting in electrostatic repulsion that hinders their adsorption by MPs [70].

Salinity neutralizes plastics’ surface charges by reducing electrostatic interactions that facilitate the sorption of other contaminants into plastics [71].

The ecological consequences of MPs on algae and marine organisms (e.g., fish, whales, corals, fur seals, and zooplankton) across various trophic levels have been highlighted in numerous studies. MPs can diminish developmental activity in tissues, reduce growth, and alter the gene expression of algal species [72].

In fish, they can determine DNA damage and neurotoxicity, interfering with the immune system, metabolic homeostasis, and oxidative and inflammatory responses [73].

MPs in over 150 fish species from marine and freshwater environments have been reported [74]. The data is critical since these fish can transfer plastic pollutants to higher-level predators, including birds and humans, favoring their bioaccumulation and toxic consequences [75].

### 3.3. Microplastics’ Impact on the Atmosphere

One significant contributor to air pollution with microplastics comes from textiles, mainly clothing, which can emit more than 1100 fibers for every gram of acrylic fabric. Studies indicate higher levels of airborne microplastics inside buildings than outdoors [76]. Activities that produce airborne MPs are putting clothing on or taking it off, drying garments, breaking down larger plastic items, and producing industrial emissions. Factors like rainfall, pollution, humidity, and particle size influence the distribution and settling of microplastics. The wind can carry MPs over long distances, polluting terrestrial ecosystems and perpetuating a cycle that worsens the issue of microplastic pollution in the environment [77].

Honey bees (*Apis mellifera*) can be bioindicators of environmental MP pollution since they can collect MPs on their wings and heads [78].

### 3.4. The Plastisphere

**Figure 4:**
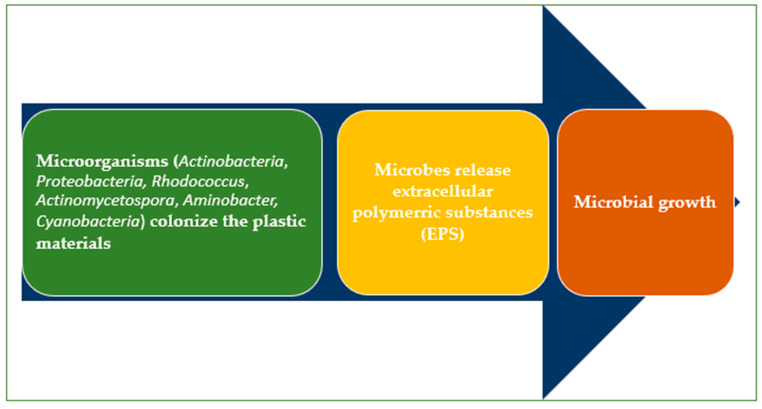
Biofilm formation on microplastic surfaces.

The plastisphere refers to the micro-ecosystem formed by microorganisms that colonize plastic surfaces. Microbes enhance further microbial attachment by producing an extracellular polymeric substance (EPS) [79,80] (Figure 4). The microbial adhesion to plastics is affected by pH, temperature, and salinity [81].

*Cyanobacteria* communities and other microorganisms (natural biofilm producers) [81,82] serve as the first microbial colonizers [83]. They produce enzymes with depolymerase activity that help to biodegrade the plastics. The initial attachment of the bacteria is influenced by the MPs’ physicochemical properties (e.g., shape, hydrophobicity, crystallinity, surface roughness, and surface charge) [84], the leaching of additives, and the establishment of an eco-corona [85].

The formation of the plastisphere can happen in terrestrial and aquatic ecosystems.

Diatoms (*Mastogloia*, *Cyclotella*, *Navicula*, *Amphora*, *Sellaphora*, *Nitzschia*, and *Pleurosigma*), algae (red, brown, and green algae), *Rhodococcus*, *Flavobacterium*, *Pseudomonas*, *Saprospiraceae*, *Planctomycetes*, *Hyphomonadaceae*, and *Erythrobacteraceae* are microorganisms that colonize the plastisphere in the marine environment along with pioneer colonizers bacterial families like *Alpha-* and *Gamma*-*proteobacteria* [61].

The microbial community in the terrestrial plastisphere closely resembles that of the aquatic plastisphere. Research has identified *Actinobacteria*, *Proteobacteria Rhodococcus*, *Actinomycetospora*, *Aminobacter,* and *Cyanobacteria* (*Nostoc* and *Scytonema*) in the terrestrial plastisphere [86], along with pioneering bacterial families like *Geodermatophilaceae*, *Beijerinckiaceae, Nocardioides*, and *Rubrobacteriaceae* [87].

## 4. Microplastics in Food

MPs are recognized as a significant emerging threat in the food sector. Their tiny size allows them to be easily absorbed by various organisms at different trophic levels and with diverse feeding strategies. Microplastic contamination is present throughout the marine food web’s five primary trophic levels. Numerous individual marine species exhibit bioaccumulation of microplastics across four primary consumer trophic levels. A meta-analysis study proved that the effect of environmental exposure to chemical additives on bioaccumulation is more significant than that of chemical additives linked to microplastics. The bioaccumulation of microplastics seems to be more closely related to the feeding strategies of marine species than to their trophic levels [88].

MP rates depend on the materials utilized in processing or storage, the techniques employed during production, and environmental contaminants [89].

MPs are discovered in plentiful amounts in seafood [90], sea salt [91], drinking water [92,93], sugar [94], meat products [95], milk [96], and honey [97,98,99,100].

MPs can contaminate tap and bottled drinking water [93]. Sunlight [99,100], the pressure and heat used during bottling operations [98], and the detergents used in washing machines that clean the bottles before filling them [101] can produce MPs.

Fibers, films, fragments, and spherules from MPs of various origins can taint sugar. This contamination may occur at multiple points in the production cycle, including processing, purification, refinement, and drying, where air currents from dryers can transport MPs [5].

The agricultural and milking processes can produce POPs [102,103]. Multilayer paper packaging can release polypropylene in milk [102].

In some honey samples, styrene, phthalates, and bisphenol A were detected, probably due to the contamination of bees during the pollination process [104].

The MPs in sea salt are attributed to the degradation of plastic materials in the ecosystem, their discharge from industrial processes, and their accumulation linked to the evaporation of seawater. An individual who consumes the daily salt intake (5 g) recommended by the World Health Organization ingests from 37 to 1000 microplastic particles yearly through sea salt [105]. Coagulation or filtration processes can be used to avoid this problem. Coagulation effectively separates solute from solvent, aggregating colloidal particles into larger flocs with coagulants until sedimentation or filtration. Nanofiltration and ultrafiltration use high pressures and large filter surface areas that limit their employment [106].

Consuming fish contaminated with MPs poses low risks for human health when the intestine is discarded [107] since the MPs accumulate in marine organisms’ digestive systems [108].

MP contamination negatively impacts terrestrial fauna. MPs enter the terrestrial food web via primary consumers, such as insects, which transfer them to higher trophic levels through predation. Birds, mammals, soil organisms, and insects can ingest MPs through contaminated food sources like plants and prey. Emerging research suggests that MP exposure may reduce reproductive success and cause developmental abnormalities in terrestrial species, impacting population dynamics and potentially leading to declines in wildlife [109]. Polypropylene and polyethylene (fibers and fragments) were found in South China livestock [110]. Plastic cutting boards and the materials used for packaging can play a role in introducing MPs into meat products [111].

## 5. Microplastic Emissions in Culinary Environments

MPs from kitchen utensils, food contact materials, packaging, and cooking methods influence the degree of food contamination.

### 5.1. Kitchen Utensil Contamination

Nonstick cookware, plastic cutting boards, and disposable cutlery are the most significant sources of MPs. Plastics’ disintegration facilitates chemical leaching and promotes the liberation of microplastics [112,113]. Heat deforms the objects and favors their release [114]. Trace elements, such as Fe^3+^, Cu^2+^, and Ca^2+^/HCO^3−^, can mitigate the emission of MPs [115].

Polyethylene MPs were found in chicken and fish prepared on polyethylene cutting boards [116,117].

Ceramic salt mills release polyethylene terephthalate, kitchen blenders discharge up to 0.78 million polypropylene MPs in 30 s [118], and dish sponges emit 100 and 200 micro-nylon and PET particles in 30 s [119].

Thus, it is recommended that glass or metal utensils be used to reduce the likelihood of MP contamination.

### 5.2. Food Packaging Contamination

The type of material used in food packaging, along with temperature and duration of contact [119,120], can lead to the release of MPs and stabilizers that can potentially cause cancer and gastrointestinal issues [121,122,123]. Food packaging can discharge harmful metals such as lead and cadmium, which have the potential to cause infertility and cancer [124]. Using biodegradable packaging such as chitosan, milk proteins, seaweed films, and glass containers reduces exposure to MPs [125,126].

The intake of MPs can vary based on factors such as human age, the type of food packaging, the specific food items consumed, cooking techniques, and airborne contaminants present while cooking.

The process of thermal expansion and contraction renders plastics more vulnerable to degradation. Cooler temperatures can cause plastics to become brittle, heightening the chances of MPs being released due to mechanical strain [127]. Hernandez and colleagues found that at 95 °C, a single tea bag can release about 11.6 billion microplastics and 3.1 billion nanoplastics into tea [128].

People who consume take-out meals approximately one to two times per week are exposed to an estimated intake of 170 to 638 MPs [129].

Baking paper and aluminum foil may emit bisphenols when used to cook acidic foods, while a reduced release is observed when they are utilized for fatty foods [130].

Transparent plastic containers release higher levels of phthalate esters than opaque ones, likely due to the need for additional plasticizers [131].

Extruded polystyrene packaging releases MPs in meat and food preparation areas [132].

The vacuum packaging process, high temperature, storage time, the chemical composition of the packaging material, and the type of food influence the contamination rate of the packaged food [133].

Paper bags made from recycled materials may contain elevated levels of phthalate esters whose migration rates are considerably greater than those observed from plastic [134]. The ink used to decorate paper can release poly-fluoroalkyl substances (PFAs) [135].

### 5.3. Cooking Methods’ Interference with Plastic Contamination

**Figure 5:**
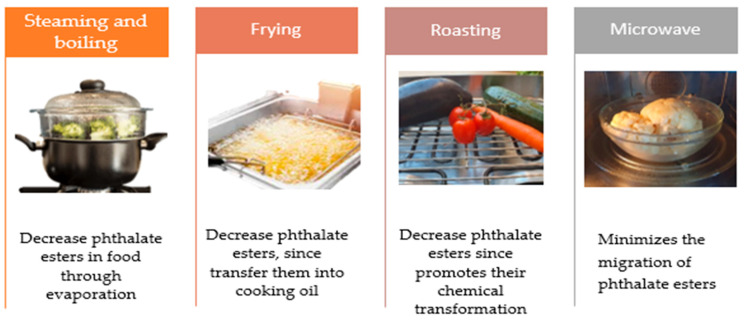
Potential food contamination with phthalate esters from containers during cooking.

Heat energy has the potential to alter the oxidation processes of plastics. It occurs primarily because the –C–C− bond within the molecular makeup of plastics can rapidly oxidize to form –C double-bond O− and –C–O− bonds under highly thermophilic conditions, thereby increasing degradation rates and improving hydrophobicity [136]. The contamination risk decreases when foods are stored at cooler temperatures. Microwaving alters the packaging material and increases the release of MPs into the foods [137]. Steaming and boiling disperse MPs into the environment through evaporation. Frying accumulates MPs in the cooking medium [137] (Figure 5).

## 6. Plastic Residues’ Impact on Human Health

Plastic residues can enter the human body, as demonstrated by their being found in feces [138]. The lack of long-term studies on the effects of micro- and nanoplastics on human health constrains our ability to assess their impact.

### 6.1. Routes of Exposure

MPs can enter our bodies when we consume contaminated foods such as seafood and salt or drink tainted water. They can also be inhaled from the air, especially indoors, where synthetic materials and dust are present, or absorbed through the skin via personal care products [139]. MPs can affect the placenta, liver, gut, lungs, heart system [140,141], thyroid, testes, and ovaries of humans [142].

Textiles, traffic exposure, industrial emissions, cigarette smoking, and construction activities can release MPs into the environment [143]. MPs resist lung degradation and can accumulate [144], causing respiratory distress and triggering cytotoxic and inflammatory responses. Moreover, MPs can contribute to interstitial fibrosis, chronic bronchitis, and asthma-like reactions [145].

Depending on their size, dosage, and individual susceptibility, NPs, bisphenol A, and phthalates can be absorbed through the derma [146]. Dermal contact with NPs can be due to the continuous release of fibers from clothes. Plastic particles that can arise from cosmetic products such as hand washes, face masks, hand cleansers, or toothpaste with a size greater than 100 nm do not get absorbed [147].

Individuals are estimated to swallow between 39,000 and 52,000 MPs annually [148], which can cause inflammatory responses, alterations in gut microbiota composition, and negative impacts on metabolic processes [149].

Following ingestion, MPs enter the enterocytes. Internalization occurs via M cells or other cells in the intestinal mucosa near Peyer’s patches through micropinocytosis, phagocytosis, and receptor-mediated endocytosis. Paracellular transport occurs through the spaces between cells, driven by differences in concentration and the size of particles. This transport is facilitated when the tight junctions are disrupted during cellular renewal and through the temporary gaps due to the epithelial injury or movement of macrophages. Intestinal macrophages and dendritic cells can directly uptake particles from the intestinal lumen [95].

### 6.2. Harmful Effects on Human Health

Micro- and nanoplastics can deteriorate organs and systems, harming organelles and membranes and affecting gene expression [150].

NPs can interact with mitochondria, the endoplasmic reticulum, and lysosomes, leading to oxidative stress and cytotoxic impacts. In in vivo tests using rats and *Eriocheir sinensis*, MPs increased the body’s production of ROS [151]. The production of reactive nitrogen species (RNS) and reactive oxygen species (ROS) is influenced by MPs’ dosage, particle size, surface characteristics, and exposure time [152]. The halogenated organic compounds and heavy metal ions adsorbed onto microplastics can exacerbate oxidative stress [153].

In vitro data suggested that polystyrene produced elevated ROS through the P62/Nrf2/Keap1 pathway [154]. MPs and NPs can disrupt the (phosphatidylinositiol3-kinase/protein kinase B) PI3/AKT signaling pathway, which determines cell apoptosis [155].

They initiate ROS-mediated P53 signaling, which activates cellular death pathways [156] and can trigger inflammation [157].

MPs and NPs contribute to metabolic syndrome by stimulating the mitogen-activated protein kinases (MAPKs) [158] and can activate the reactive oxygen species/transforming growth factor-β/Smad (ROS/TGF-β/Smad) pathway, causing fibrotic damage [137].

Finally, they can promote carcinogenesis by activating the nuclear factor erythroid 2-related factor (Nrf2) [135,158].

MPs cross the blood–brain barrier, negatively affecting neurological functions and impairing memory and learning. They increase the levels of harmful substances such as malondialdehyde and reactive oxygen species (ROS) and reduce the levels of glutathione and acetylcholine, which are essential for cognitive performance [159].

Exposure to traffic pollution can determine mild cognitive impairment and can increase the risk of developing Alzheimer’s disease [147].

Additionally, MPs promote microthrombosis and neuronal cell apoptosis, decreasing the expression of connexins within the blood–brain barrier [160].

Prolonged exposure to MPs disrupts the tight junctions between lung cells, compromising lung barrier integrity [161,162].

MPs can absorb heavy metals (e.g., cadmium), which are liver-toxic [163]. The consequent liver fibrosis [164] and steatosis can determine glucose metabolism disorders [165].

Dietary exposure to MPs reduces mucus secretion, promotes epithelial cell apoptosis, and increases intestinal permeability [166]. MPs impact the richness and diversity of the gut microbiome (they increase *Staphylococcus* and reduce *Parabacteroides* levels) [167].

The kidney serves as a primary site for the MPs accumulation. MPs can promote the generation of reactive ROS [168], which intensify endoplasmic reticulum stress and induce inflammation and renal damage [169].

MPs can inflame the testes, disturb the testicular blood barrier, and induce inflammatory pathways that attenuate sperm count and motility and increase malformations [170].

In females, they cause ovarian inflammation and lower the extrusion rate of polar bodies and oocyte quality, along with apoptosis of granulosa cells and the fibrosis of the uterus, plausibly resulting in infertility in females [170].

Assessing dose–response relationships for microplastics is challenging, as they may be independent of microplastics themselves due to additives that may leach from them and chemicals they have absorbed. Microplastics found in the environment are usually smaller than 5 mm, yet studies often target a narrow size range of 0.5 to 50 µm, restricting the exploration of their toxicological impacts. The differences in size, composition, and type of microplastics further hinder the ability to determine a threshold for their harmful effects. Additionally, most research has focused on the impact of polystyrene microplastics on higher mammals, resulting in a substantial knowledge gap regarding other types [171].

## 7. Analytical Methods Used to Detect Plastic Residues

**Figure 6:**
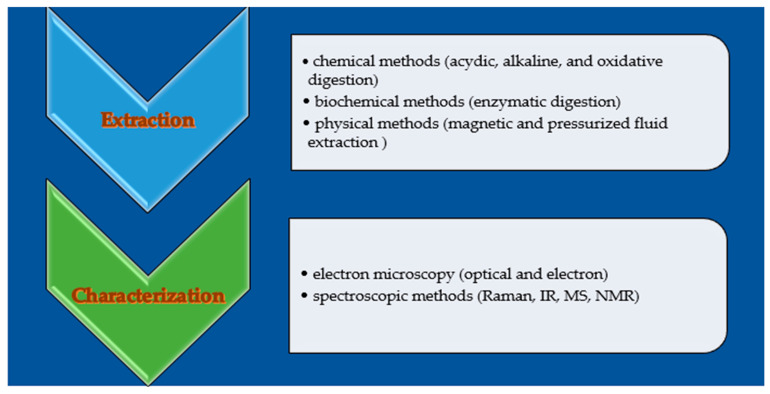
Analytical methods used to evaluate the plastic residues in foods.

The analytical protocols to determine MPs and NPs in foods involve chromatographic methods to eliminate the matrix interferents and microscopy or spectroscopic apparatuses to identify and quantify them (Figure 6) [172]. The lack of agreement regarding the essential information required for safety assessments complicates identifying what should be measured and documented [173]. The lack of standardized methods and consistent measurement units creates significant challenges in assessing the distribution and composition of microplastics (MPs). This inconsistency hampers our ability to accurately analyze their presence and impact, making it challenging to develop effective mitigation and environmental protection strategies [173].

### 7.1. The Extraction of Microplastics from Biological Specimens

A cleanup procedure must not affect MPs’ integrity and must eliminate the contaminants to allow a precise identification and dosage of MPs.

Chemical (alkaline, acidic, or oxidative digestion), physical (pressurized fluid extraction or magnetic separation), or enzymatic methods can be employed to extract microplastics from biological samples. Chemicals can damage microplastics, so physical or enzymatic procedures are preferable.

FTIR and Raman spectroscopies are employed to evaluate the effects of digestion on the mass, morphology, and surface area of the polymer particles [174].

Alkaline, acidic, and oxidative digestion differ in cost and accessibility. Alkaline digestion is widely accessible due to its simplicity. It is favored for its low cost and efficiency in removing organic material while preserving microplastic integrity. Acidic digestion is moderately accessible but requires careful handling. It may degrade some plastics, affecting cost-effectiveness. Oxidative digestion is the least accessible, needing controlled environments and expertise. It requires specialized equipment, raising costs [175].

Alkaline digestion is a hydrolytic method that employs NaOH or KOH (KOH is the most effective agent) to break down tissues. Alkaline agents can degrade PET (more NaOH than KOH) [172]. The effectiveness of alkaline digestion decreases when applied to water and sediment samples, mainly due to the presence of biogenic organic materials from plant sources, including woody debris, leaves, and algae, along with components from carapaces and shells [176] that contain complex compounds like cellulose, lignin, hemicellulose, humic substances, tannins, and chitin, which are poorly digested [177,178,179,180].

Nitric acid is widely employed for acidic digestion. It can degrade proteins, lipids, and other organic materials of biological tissues [181]. Some of the protocols use a 3:1 mixture of nitric and hydrochloric acids. This mixture has the added advantage of improving the degradation of organic substances and aiding in eliminating some inorganic materials like calcium carbonate that are likely to occur in the samples. In addition, the combination of nitric and perchloric acid in a 1:1 ratio is sometimes employed. This combination is quite effective because perchloric acid is a powerful oxidizer that disassembles intricate organic structures and ensures complete separation of MPs from the biological matrix [182]. Peroxymonosulfuric acid, produced by combining hydrogen peroxide with sulfuric acid, is an oxidizing agent significantly stronger than nitric acid. It is employed to eliminate large quantities of organic contaminants in studies on lake sediment pollution and river sediments [177].

Oxidative digestion employs hydrogen peroxide with a ferric ion (Fe (II)) (Fenton’s reagent) or sodium hypochlorite (NaClO) [174] to break complex compounds into carboxylic acids, carbon dioxide, aldehydes, and water under relatively mild conditions. It is followed by density separation or filtration [183].

Pressurized fluid extraction (PLE) employs solvents under subcritical temperature and pressure conditions to recover semi-volatile organics from solid materials. PLE can be combined with scanning electron microscopy, visual inspection, micro-Fourier-transform infrared spectroscopy, and GC coupled to tandem mass spectrometry to detect MPs [183]. In the initial extraction phase, semi-volatile organics are removed using methanol at 100 °C. The recovery of the MPs from the residual matrix is obtained using dichloromethane at 180 °C [184].

Dynamic PLE requires a specialized high-pressure pump to control the solvent flow rate accurately, along with solvent preheating coils and a back pressure regulator, as opposed to a simple open/close valve commonly found in static systems [185].

Magnetic separation consists of binding sustainable, cost-efficient, and low-toxic magnetic nanoparticles to MPs to allow their separation by applying magnets [186]. The magnetic separation method does not impose a minimum size restriction for the MP separation, as the size dependency can be adjusted by changing the size and type of magnetic nanoparticles used. Hydrophobic and electrostatic interactions are used as driving forces. Hydrophilic magnetic nanoparticles separate MPs using hydrogen bonds. In contrast, hydrophobic nanoparticles employ electrostatic attraction [187]. Magnetic nanoparticles are employed to collect polyethylene, polyethylene terephthalate, polypropylene, polyamide, polystyrene, and polyvinyl chloride [187].

Enzymatic digestion employs enzymes, such as proteinase, cellulase, lipase, or chitinase, to eliminate organic matter. Due to its selective nature, enzymatic hydrolysis reduces the risk of modifying microplastics, which occurs with chemical hydrolysis [188].

### 7.2. Plastics Characterization

Plastics characterization can be performed by microscopy and spectroscopy (Table 3).

The characterization performed by optical and electron microscopy provides information about MP size, distribution, morphology, thickness, topography, state of degradation, and color. Optical microscopy cannot resolve submicron particles; instead, the electron microscope, whose resolution range is about 1 nm and whose field of view is approximately 1 mm^2^, succeeds [189].

IR spectroscopy, Raman scattering, and Fourier transform infrared (FT-IR) spectroscopy yield distinctive spectra crucial for identifying and characterizing plastics. Raman spectroscopy exhibits heightened sensitivity to nonpolar symmetric bonds, whereas FT-IR is more adept at identifying polar functional groups [190]. Both techniques exhibit high detection limit values (approximately 20 µm) that can be further reduced (to approximately 1 µm) if imaging is used. Nevertheless, NPs measuring less than 1 µm can be overlooked, resulting in an underestimation of microplastic concentrations in food. Surface additives can also interfere with the estimation of microplastics [191].

Various techniques, including gas chromatography-mass spectrometry (GC-MS), liquid chromatography–tandem mass spectrometry (LC-MS/MS), time-of-flight mass spectrometry (ToF-MS), atmospheric solids analysis probe mass spectrometry (ASAP-MS) [192], isotope ratio mass spectrometry (IR-MS) [193], and single particle–inductively coupled plasma mass spectrometry (SP-ICP-MS), are employed to analyze MPs and NPs in food matrices [194]. Each methodology has varying degrees of accessibility and cost-effectiveness. GC-MS and LC-MS/MS techniques are commonly found in food safety laboratories, making them relatively accessible. Py-GC–MS is considered a technique of choice for analyzing MPs. These methods offer outstanding sensitivity but require expensive equipment and highly trained personnel. ToF-MS and ASAP-MS are not as frequently used due to the specialized nature of the equipment needed. These techniques allow for rapid analyses but can be costly, especially in high resolution. IR-MS and SP-ICP-MS are utilized in environmental and regulatory labs. Their accessibility is not widespread. These methods excel in isotopic and elemental analysis, although the instruments are expensive, and maintenance can be quite high [195].

A key challenge in MPs analysis is their complex composition, often involving multiple polymers that can co-pyrolyze, affecting yield and quantification accuracy. Research on polymer cross-interference during pyrolysis is limited. Diluting reference materials is more manageable for solutions than solids [196].

NMR provides precise 3D structural information based on molecular vibrations while preserving sample integrity. NMR is used with little frequency to characterize higher-molecular-weight compounds due to the complexity of the spectra. Polyvinyl chloride, polyethylene, polystyrene, polyethylene terephthalate, and polyamide were studied using NMR [197].

**Table 3:** Comparisons among different detection methods.

Method	Detection Limit	Features	Advantages	Disadvantages	References
Optical microscopy	50 μm	Quantification; non-destructive	Rapid and cost-effective identification of particle characteristics such as size, color, shape, and surface structure.	Major inaccuracies may occur, requiring a considerable amount of time; errors in identification.	[189,198]
Electron microscopy	1 nm	Quantification; non-destructive	Electron microscopes can achieve resolutions up to 0.2 nanometers.	Preparing samples for electron microscopy can be time-consuming and may alter the specimen, potentially introducing artifacts.	[189,198]
FTIR	10 μm	Identification; non-destructive	Non-destructive. It has a spectrum database. It can detect several thousand particles with a single measurement. Reliable outcomes, exceptional sensitivity, rapid screening capabilities, and eco-friendliness.	It is costly and requires skilled operators. Pretreatment is essential. It is labor-intensive and can be easily affected by water. Sample preprocessing, organic soil matter, and the age of microplastics may also alter the chemical bonds of the samples.	[190,191,198]
Raman	1 μm	Identification; non-destructive	Non-destructive nature; capable of identifying additives. Excellent spatial resolution, great precision, high sensitivity, exceptional specificity of fingerprint spectrum, and no harm to the sample, with no need for specific sample thickness. It has a spectrum database.	The spectral library is not as extensive as FTIR and may require a significant amount of time. Additives and impurities can lead to interference. Adequate sample preparation is essential. Choosing the appropriate wavelength is vital for reducing sample fluorescence and obtaining a strong signal.	[190,191,198]
MS	The lowest detectable limit for PC is 27.7 µg/kg, while for PET is 178.3 µg/kg	Identification and quantification; destructive	It offers an effective detection limit, facilitates high-volume sample input, and operates quickly and automatically. Can measure additives.	Needs pretreatment.	[192,193,194,198]
NMR	0.2~10 µg/mL	Identification and quantification; non-destructive		NMR is infrequently employed for the characterization of higher-molecular-weight compounds because of the intricate nature of the spectra.	[197]

## 8. Plastic Waste Management

Plastic waste will reach 265 million tons annually by 2060 [199]. Key plastic challenges arise from their long degradability, treatment expenses, and environmental problems that occur in poor recycling. The basic circular economy principle can be adopted to overcome this challenge. The circular economy promotes repairing, upgrading, and redistributing second-hand goods. This concept is reduced to the 3Rs—reduce, reuse, recycle. With the implementation of this circular economy model, it is anticipated that longevity will be given to waste that can be turned into resources of great value for further use [200]. Gunter Pauli coined the term “upcycling” to refer to waste conversion (by depolymerization, polymerization, and functionalization) into polymers, molecules, and materials [201,202,203]. Upcycling occurs when plastic waste is reused without diminishing its quality or functionality for subsequent applications [201]. Upcycling of plastic waste can be used in the production of high-performance fuels (e.g., hydrogen and liquid alkanes) [204] and carbon materials (e.g., carbon dots [205], nanofibers [206], nanosheets [207], microspheres [208], three-dimensional porous carbon [209], graphite, graphene [210], and carbon nanotubes [211]). For example, polycarbonate (PC) can be made by repolymerizing bis (hydroxyethyl ether) [212], fiberglass-reinforced plastic by the depolymerization of PET [213], and cyclic carbonates and carbamates by the depolymerization of BPA-PC [214]. Erickson et al. examined the economic and environmental advantages of establishing an upcycling infrastructure in the continental United States to produce low-density polyethylene and polypropylene from post-consumer plastic waste. They suggested that this infrastructure could create a market valued at nearly USD 20 billion annually, using a computational framework that combines techno-economic assessment, life-cycle evaluation, and value chain optimization [215].

Primary (closed loop), secondary (mechanical), tertiary (chemical), and incineration methods can be employed to convert plastic waste into reusable materials (Table 4). Only primary recycling produces high-quality plastic; the others give lower-quality plastic, often called “downgrading” or “downcycling”, since high temperatures and mechanical stress degrade the polymers [216].

Mechanical recycling is utilized for PET and PE plastics. Recycled plastics have lower quality than virgin plastics [201]. Recycling is not possible for heavily contaminated or multi-material plastics. This process includes sorting and cleaning the plastic waste, producing pellets, and heating it before making new products. Mechanical recycling may release volatile organic compounds into the environment [216].

Thermochemical and catalytic processes (e.g., pyrolysis, gasification, hydrocracking, and depolymerization) transform plastic waste into fuels or monomers. Chemical recycling is more adept at processing mixed or contaminated plastics than physical recycling [217,218,219].

Biodegradation is a nontoxic approach that can transform plastic by using enzymes. Ongoing efforts are directed towards developing strains with improved hydrolytic activity against plastic polymers by altering amino acid sequences at active sites through genetic techniques. Significant areas of investigation involve transcriptional regulators and how to enhance the enzymes’ ability to degrade specific polymers. Only a few studies have examined how the modifications affect the enzymes’ specificity [220,221,222].

## 9. MPs Legislation

MPs present a significant and persistent threat to human health and the environment. Their long-term effects could negatively impact future generations worldwide. Microplastic regulations vary across regions, reflecting the differences in environmental priorities and regulatory approaches. The European Union (EU) has notably advanced in this domain. The EU aims to accomplish a 30% decrease in MPs emissions by 2030 by implementing bans, improved waste management practices, and stricter regulations [223]. On 25 September 2023, the European Commission approved Regulation (EU) 2023/2055 limiting the intentional incorporation of microplastics in products. This initiative aimed at reducing the release of plastic pellets into the environment, along with its associated Impact Assessment (IA), arises from the Commission’s pledge to address the unintended discharge of microplastics [224].

Three key principles were identified to standardize legislation on MPs and pave the way for a sustainable and environmentally responsible future [225]:

1. Precautionary Principle (PP): This principle, embedded in environmental legislation (European Union, European Treaty, Article 191; REACH CE 1907/2006) [226], advocates for preventive measures in risk management, particularly in the absence of scientific consensus, while also promoting a circular economy.

2. Principle of Proportionality (PrP): It is defined in the European Union’s European Treaty, Article 5 [227], which mandates necessary actions to support the Precautionary Principle in mitigating environmental risks.

3. Polluter Pays Principle (PPP): As outlined in the European Union, European Treaty, Article 191 [228]. This principle holds producers and consumers responsible for the costs associated with reducing MP pollution, thereby fostering environmental accountability and the creation of sustainable products.

REACH regulation is applied to monomers and polymers. However, polymers are currently exempt from the initial registration and evaluation phases, which are essential to REACH. The European Union does not differentiate between new and existing polymers, meaning all are exempt from these requirements until it becomes feasible to identify those that pose potential risks to human health or the environment. Instead, if a polymer contains more than 2% of a monomer and the annual usage exceeds 1 ton, the importer or producer must register the monomer with the European Chemical Agency [229].

Postle et al. [230] and De Toni et al. [231] investigated the economic viability of registering polymers under the REACH regulation for the European Commission.

Postle et al. [230] categorized polymers into two primary groups: those with identical constituents and those with varying constituents. Polymers with identical constituents are further divided into three scenarios: structurally identical polymers, polymers classified as a single substance under the Dangerous Substances Directive, and polymers with incremental and consistent changes. In contrast, polymers with different constituents are characterized by variations in counter-ions and changes involving similar monomers [230]. De Toni et al. assessed when a polymer can be identified as a “Polymer of Low Concern” (PLC), considering factors such as cationic nature, molecular weight, oligomer content, and the availability of data on human and environmental hazard classifications under the EU CLP regulation [231].

Article 5 of the Treaty on European Union states the mandates that measures addressing environmental risks must be necessary and proportionate, preventing both excessive and insufficient responses [225].

Finally, Article 191 of the European Treaty proposes three Extended Producer Responsibility models.

The “Buy-Back Depository Mechanism” incentivizes consumers to return plastic products by offering buy-back prices at collection points like reverse vending machines [232].

The second model forces the producers, importers, and brand owners to contribute to a fund based on the amount of plastic they market.

The third model sets specific recycling targets for producers according to their plastic volumes, supported by a “Plastic Credit” system that validates recycling efforts [233].

The U.S. has focused on promoting biodegradable and recyclable materials; implementing policies to decrease plastic waste; enhancing recycling and disposal systems; strengthening cleanup efforts for plastic pollution; and preventing microplastic contamination in waterways and oceans [234]. The U.S. Geological Survey (USGS) provided data for environmental decisions, building coalitions for collaborative research, and creating standardized MP sampling protocols [235].

Asian nations have adopted strategies shaped by their economic development, environmental priorities, and regulations (ASEAN Regional Action Plan (2021–2025)) [236], improved the regional cooperation (see ASEAN-Norway, which involves collaboration between ASEAN and Norway [237], and ASEAN+3 Marine Plastic Debris, which includes ASEAN Member States and the People’s Republic of China, Japan, and the Republic of Korea [238]), and encouraged responsible waste disposal among residents and tourists by employing public awareness campaigns. In Indonesia, 2012 waste-bank initiative incentivized households to sort waste [239]. Innovative technologies, such as the ocean cleanup project, are being used to eliminate plastics from Southeast Asian waterways [240]. Japan and South Korea enforce strict rules on microplastics in cosmetics and industry [241], while China focuses on managing plastic waste and banning certain single-use plastics [242]. In Southeast Asia, countries like Indonesia and Malaysia, heavily impacted by plastic pollution, are implementing community cleanups and seeking policy reforms [243].

## 10. Materials and Methods

### 10.1. Search Strategy

**Figure 7:**
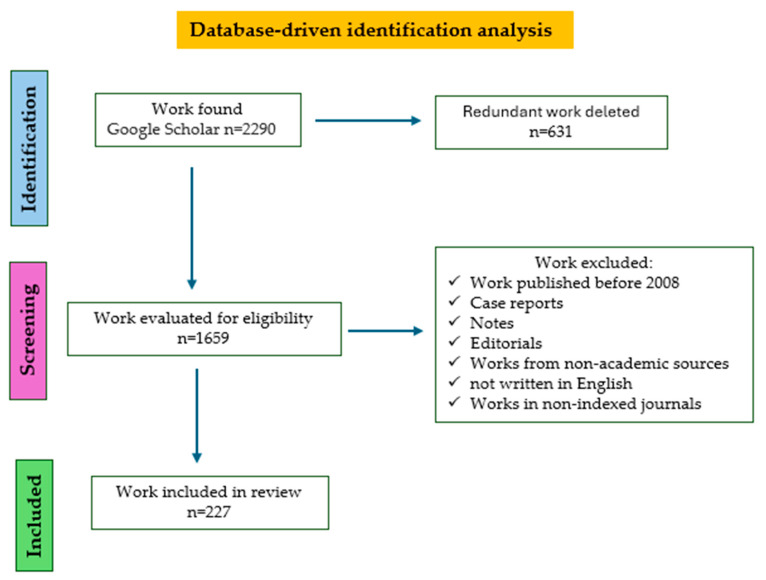
Flowchart representation of this study using PRISMA.

The PRISMA (Preferred Reporting Items for Systematic Reviews and Meta-Analyses) guidelines were used to guarantee the validity and strength of the research outcomes [244]. Google Scholar databases were employed as information sources (Figure 7).

### 10.2. Inclusion Criteria

The criteria for eligibility encompassed research articles and reviews on the following topics.

- Food toxicology: Plastic and microplastic residues in foods and a source of contamination of microplastic residues in foods.
- Analytical methods to detect microplastic residues in foods.
- Technological approaches and sustainable strategies to address the challenge of microplastics in foods.

The criteria for exclusion encompassed articles not written in English and publications originating from non-academic sources.

Literature papers published between 2008 and 2024 were discussed in this work.

The search terms utilized included the following: “microplastics in foods”, “cooking methods” microplastics; microplastic “waste recycling”; “nanoplastics toxicity”; “circular economy” microplastic. A total of 244 articles were reviewed.

Papers excluded were works published before 2008, case reports, editorials, notes, works from non-academic sources, works in non-indexed journals, and non-English articles.

## 11. Conclusions

This review provides a comprehensive overview of the composition, classifications, characteristics, sources, and ecological effects of plastics and their by-products, emphasizing the potential dangers to human health when they infiltrate the food chain. The literature review indicated that humans are exposed to MPs and NPs, and the long-term toxicological impacts remain unknown. Therefore, it is vital to improve public engagement in managing plastic waste to prevent it from entering ecosystems and causing harm until more effective waste recovery methods can be implemented. Further investigation is crucial into the interactions between micro- and nanoplastics and human cells, their effects on DNA, and the long-term health implications. In addition, identifying new biomarkers related to exposure and creating and validating innovative detection techniques for MPs and NPs in environmental and food samples would benefit public authorities and analytical laboratories. Finally, creating advanced technologies that can efficiently decompose microplastics into reusable chemical components and discovering enzymes or microorganisms that can convert microplastics into valuable materials like bioplastics or construction resources could significantly reduce environmental pollution.

## Figures and Tables

**Table 1:** Plastics application overview.

Plastics	Short	Applications	Reference
Synthetic Plastics			
Polyethylene	PE	Food packaging, milk and water bottles, plastic bags, toys	[14]
Polypropylene	PP	Fibers, sheets, strapping, films, injection molding	[15]
Polyvinyl chloride	PVC	Packaging, healthcare applications, building construction, transportation, electrical application	[16]
Polystyrene	PS	Packaging, kitchen appliances, toys, computers	[17]
Polyethylene terephthalate	PET	Textiles, bottles, fibers, films	[18]
Polyurethane	PU	Automotive industry (paint or polyurethane-based coatings), catheters, surgical drapes, hospital beddings, wound dressing, textiles	[19]
Bioplastics			
Polyhydroxyalkanoates	PHA	Packaging, bioremediation, 3D printing, biomedical use	[20]
Poly(lactic acid)	PLA	Packaging, agriculture 3D printing, biomedical use	[21]
Poly(butylene adipate-*co*-terephthalate)	PBAT	Packaging, single-use catering items, textile industry, horti- and agriculture	[22]
Poly(butylene succinate)	PBS	Packaging, disposable tableware, biomedical use, agriculture (release of fertilizers and pesticides), fishery	[23]
Starch, thermoplastic starch	TPS	Injection-molded thermoformable flat films	[24]
Bio-poly(ethene terephthalate)	Bio-PET	Textile fibers, fisheries, parachutes, horticulture, musical instrument strings, tennis rackets	[25]
Bio-poly(propene)	Bio-PP	Electrical devices, concrete additives, packaging materials, textile fibers, automotive parts	[25]
Poly(ε-caprolactone)	PCL	Biomedical use, packaging	[26]
Cellulose acetate	CA	Eyeglasses frames, artificial silk, cigarette filters	[27]

**Table 2:** Comparative analysis of plastic breakdown processes.

Process Type	Condition	MP Type	Performance %	Degradation Time	Reference
Photodegradation	UV light +TiO_2_	polystyrene	4.65	50 h	[43]
	Visible light + ZnO nanorods	polypropylene	65	456 h	[43]
	Au@Ni@TiO_2_	polystyrene	67	40 s	[43]
Thermal degradation	Gasification in supercritical water (800 °C)	polycarbonate	50.8	60 min	[43]
Biodegradation	*Bacillus* sp. *YP1 in* liquid carbon-free medium with 1 g of polymer	polyethylene terephthalate	10.7	2 months	[43]
	*Trichoderma harzianum* in mineral salt medium	polyethylene	40	3 months	[43]
	*Aspergillus tubingensis* in mineral salt medium	polyester polyurethane	90	0.75 months	[43]

**Table 4:** Comparisons among different recycling and upcycling technologies.

Method	Advantages	Disadvantages	References
Mechanical recycling	Rapid and cost-effective.	Requires initial sorting and reduces mechanical efficiency. It releases volatile organic compounds into the environment. It does not process heavily contaminated or multi-material plastics.	[201,216]
Biodegradation of polymers	Eco-friendly. It operates under mild conditions. Product yield is low.	The yield of the product is low.	[201,217,218,219]
Pyrolysis It was carried out in the absence of oxygen atmospheric pressure and 400–800 °C.	It can process mixed or contaminated plastics. Common polymers break down when exposed to elevated temperatures.	The resulting products are complex.	[201]
Gasification It employs elevated temperatures compared to pyrolysis in an oxygen-rich environment.	It generates valuable gas mixtures, commonly known as syngas, which can be used to create fuel or incinerated directly for energy production.	It is quite sensitive to impurities.	[201]

## Data Availability

Data sharing is not applicable to this article as no datasets were generated or analyzed during the current study.
